# Characterization of Conventional One-Step Sodium Thiosulfate Facilitated Gold Nanoparticle Synthesis

**DOI:** 10.1186/s11671-015-0940-1

**Published:** 2015-05-28

**Authors:** Scott-Eugene Saverot, Laura M Reese, Daniela Cimini, Peter J Vikesland, Lissett Ramirez Bickford

**Affiliations:** Department of Biological Systems Engineering, Virginia Tech, Seitz Hall, Blacksburg, VA 24061 USA; Department of Biomedical Engineering and Mechanics, Virginia Tech, 325 Stanger Street, 310 Kelly Hall (MC 0298), Blacksburg, VA 24061 USA; Virginia Tech Center for Sustainable Nanotechnology, Virginia Tech, Kelly Hall, Blacksburg, VA 24061 USA; Department of Biological Sciences and Virginia Bioinformatics Institute, Virginia Tech, 1015 Life Science Circle, Blacksburg, VA 24061 USA; Department of Civil and Environmental Engineering, Virginia Tech, Durham Hall, Blacksburg, VA 24061 USA; Department of Mechanical Engineering, Virginia Tech, Kelly Hall, Blacksburg, VA 24061 USA

**Keywords:** Gold-gold sulfide, Nanoplates, Photothermal therapy, Theranostic nanoparticles

## Abstract

Gold-gold sulfide nanoparticles are of interest for drug delivery, biomedical imaging, and photothermal therapy applications due to a facile synthesis method resulting in small particles with high near-infrared (NIR) absorption efficiency. Previous studies suggest that the NIR sensitivity of these nanoparticles was due to hexagonally shaped metal-coated dielectric nanoparticles that consist of a gold sulfide core and gold shell. Here, we illustrate that the conventional synthesis procedure results in the formation of polydisperse samples of icosahedral gold particles, gold nanoplates, and small gold spheres. Importantly, through compositional analysis, via UV/vis absorption spectrophotometry, transmission electron microscopy (TEM), and energy dispersive x-ray spectroscopy (EDS), we show that all of the nanoparticles exhibit identical face center cubic (FCC) gold crystalline structures, thus suggesting that sulfide is not present in the final fabricated nanoparticles. We show that icosahedrally shaped nanoparticles result in a blue-shifted absorbance, with a peak in the visible range. Alternatively, the nanoplate nanoparticles result in the characteristic NIR absorbance peak. Thus, we report that the NIR-contributing species in conventional gold-gold sulfide formulations are nanoplates that are comprised entirely of gold. Furthermore, polydisperse gold nanoparticle samples produced by the traditional one-step reduction of HAuCl4 by sodium thiosulfate show increased *in vitro* toxicity, compared to isolated and more homogeneous constituent samples. This result exemplifies the importance of developing monodisperse nanoparticle formulations that are well characterized in order to expedite the development of clinically beneficial nanomaterials.

## Background

Gold-based nanoparticles have received immense research attention due to their unique optical properties, biocompatibility, and ease of surface modification [1]. Careful manipulation of the resonance oscillation of conductive electrons, or surface plasmon resonance (SPR), enables these particles to be utilized in an array of biomedical applications including photothermal therapy and imaging [2]. Gold nanocages, nanorods, nanoshells, and nanoplates have all been investigated due to the location of their SPR in the near-infrared (NIR) region, where tissue attenuation is minimized and light penetration is maximized [3]. One specific formulation of nanoparticles of particular interest is gold-gold sulfide nanoparticles (GGS NP). To date, GGS NP have been investigated in pre-clinical studies spanning chemotherapy delivery to tumors [4] to immuno-targeted cancer therapy and imaging [5–8]. GGS NP are reportedly advantageous over other gold formulations due to their rapid and facile one-step synthesis, superior NIR absorption efficiency (98 %) and optimal size for accumulation within tumors *in vivo* [5]. In spite of their demonstrated potential and compelling characteristics, GGS NP have not reached the level of human clinical testing seen with other gold moieties, such as gold nanoshells (NCT00848042, NCT016794) or gold colloid (NCT0035690). Initially, GGS NP were proposed as having an icosahedral, core-shell nanostructure [9, 10]. Subsequent studies have disputed this, asserting that the particles are actually gold-aggregates or pure gold nanoparticles [11–16, 6]. Based on the original published synthesis procedures, GGS NPs can be characterized as having two SPR bands: a 520 nm peak attributed to small spherical gold nanoparticles and an 800 nm peak contributed by a heterogeneous combination of predominately icosahedra, nanoplates, and irregularly shaped asymmetric nanoparticles [6]. To realistically receive approval from the Food and Drug Administration (FDA) for ultimate *in vivo* applications, nanoparticle solutions must be homogenous and well characterized to ensure consistent particle performance [17]. This would facilitate accurate prediction of the biodistribution and ultimate fate of the nanoparticles, which is necessary for identifying effectiveness as well as potential health hazards and long-term toxicity. In an effort to maximize monodispersity, and thus increasing their clinical potential, recent alternative synthesis methods have been developed including: synthesis of Au_2_S cores from H_2_S gas and potassium dicyanoaurate followed by pure gold shell growth, synthesis with an alternate gold precursor and radiation-induced reduction, and a two-step synthesis method that resulted in increased nanoplate formation [18–20]. Additional efforts have focused on eliminating small spherical gold impurities (with an SPR absorbance at ~520 nm) using physical methods such as filtration and dialysis [21, 22]. While these distinct fabrication procedures are in various stages of research and development with their own advantages, we proceed with a detailed analysis of conventional GGS NP fabrication to clarify unknown issues for potential future nanomedicine applications, namely, a thorough characterization and elucidation of the structure of these nanoparticles and their elemental composition, confirmation of the nanoparticulate species that predominately contributes to the NIR peak, and a closer examination of sample polydispersity and cellular toxicity. This will lead to future studies aimed at creating optimal monodisperse solutions in order to minimize undesirable toxicity [17, 23].

## Methods

### Gold-Gold Sulfide Nanoparticle Synthesis

Nanoparticles were synthesized based on previously described methods using conventional fabrication procedures [5, 7]. Briefly, 2 mM hydrogen tetrachloroaurate (III) trihydrate (HAuCl_4_:3H_2_O, Sigma Aldrich) and 1 mM sodium thiosulfate (Na_2_S_2_O_3_, Sigma Aldrich) were prepared in Milli-Q water in amber bottles and allowed to age for 3 days prior to synthesis. Na_2_S_2_O_3_ was added to the HAuCl_4_ in a round bottom flask under mild stirring at room temperature (25 °C) and allowed to react for 1 h at a volumetric ratio of 1.03:1, forming the GGS NP. For temperature studies, the nanoparticles were similarly prepared but stirring occurred on either a hot plate (50 °C), under refrigeration (4 °C), or on ice (0 °C). All nanoparticle samples were centrifuged twice at 3200 *g* for 40 min, and pellets were resuspended in H_2_O to a final optical density (OD) of 1.3. Particles were PEGylated by mixing 1 mL of 250 mM PEG-SH (Laysan Bio 2000 MW) in ultrapure water with 9 mL of the nanoparticle suspension on ice, followed by constant agitation overnight at 4 °C (Rotoflex). PEGylated particles were centrifuged at 3200 *g* for 40 min at 10 °C to remove excess PEG and were stored in the refrigerator at 4 °C until further use.

### Nanoparticle Characterization

UV/vis spectrophotometry analysis was performed using a Cary 60 UV/vis spectrophotometer (Agilent). For transmission electron microscopy (TEM) analysis, 5 μL of nanoparticle solution (1 mg/mL) were drop cast onto a carbon 200-mesh copper grid (Ted Pella) and the grids were covered and air-dried overnight, prior to imaging. TEM imaging was performed with a JEOL 2100 field thermionic emission TEM equipped with a silicon-drifted detector-based energy dispersive x-ray spectroscopy (EDS) system. ImageJ software was utilized to determine nanoparticle yields. Selected area electron diffraction (SAED) patterns were obtained using the field-limiting aperture, and fast Fourier transforms (FFT) were obtained in high-resolution mode. Determination of the 3D structure of the nanoparticles was obtained using tilt tomography and a tilt angle of 30°. DigitalMicrograph (Gatan) was used to analyze diffraction patterns, for FFT, to measure d-spacing, and to determine particle sizes.

### Growth Kinetics

To evaluate the nanoparticle growth kinetics using conventional formulations, nanoparticles were synthesized in a microwave to accelerate nucleation. HAuCl_4_ and Na_2_S_2_O_3_ were mixed and microwaved for 10 s at 450 W power to induce nuclei formation and then immediately quenched on ice to prevent further nanoparticle growth. As a control, another subset of nanoparticles was microwaved under identical conditions and allowed to react at room temperature for 1 h.

### Seed-Mediated Growth

A commonly used approach for nanoparticle synthesis that isolates nucleation from growth is a seed-mediated method. Small monodisperse nuclei are formed and then allowed to react with additional metal salt and a reducing agent to form larger particles of consistent sizes. Gold seeds were formed based upon methods previously described by Haiss et al. [24]. Briefly, 1 mL of 1 wt% HAuCl_4_ was first added to 90 mL Milli-Q H_2_0, followed by the addition of 2 mL of 38.8 mM sodium citrate while the solution stirred for 2 min. Fresh 0.075 wt% NaBH_4_ was then added drop wise until the solution turned a red wine color (indicative of seed development), followed by stirring for five additional minutes. Next, 2 mL of 1 mM Na_2_S_2_O_3_ were mixed with 0.25 mL of gold seed and aged for 24 h. 1.75 mL of 2 mM HAuCl_4_ was added to the solution of gold seed and Na_2_S_2_O_3_ while stirring and allowed to react for 1 h at room temperature.

### Size-Selective Separation of Polydisperse Nanoparticles

Two different methods for nanoparticle size separation were used: density gradient separation and microfiltration. Density gradient separation was based upon previous methods, wherein 2 mL gradient glycerol concentrations (ranging from 30–90 % of glycerol by mass) were first layered in a 15-mL conical tube [25]. Next, 0.8 mL of bare particles were aliquoted on the topmost layer, and the samples were centrifuged for 40 min at 3200 *g* at 40 °C. Distinct bands of nanoparticles were extracted with a dispensing needle, centrifuged at 4722 *g* for 8 min to remove the glycerol, and resuspended in water. For microfiltration, PEGylated nanoparticles were sequentially passed through 0.22-, 0.1-, and 0.05-μm filters (Polycarbonate, Sterlitech). The 0.22- and 0.1-μm filters (PVDF Millex) were sonicated in a water-filled beaker for 30 min to remove the captured nanoparticles. Nanoplate concentration in different samples was determined by physically counting the number of nanoplates out of 100 particles in the field of view via TEM imaging.

### Nanoparticle Stability

Prior to performing toxicity studies, PEGylated nanoparticles were assessed for stability. Nanoparticles were suspended in DMEM-F12 media at an OD of 0.66. Stability was monitored every 30 min over 24 h using the scanning kinetics function of the UV/vis spectrophotometer. The absorbance at the NIR resonant peak was normalized over time. A significant decrease in absorbance reflects nanoparticle instability due to protein absorption and the settling of the particles [26].

### Nanotoxicity Studies

To evaluate potential undesirable toxic effects of the nanoparticles towards healthy cells, PEGylated nanoparticles were incubated with hTERT-RPE-1 (RPE-1) cells, an immortalized human telomerase reverse transcriptase-retinal epithelial cell line. GGS NP have formerly undergone testing for photothermal therapy of solid tumors, which develop within epithelial tissues. RPE-1 cells represent an ideal model for toxicity tests, given that epithelial cells are the most abundant healthy cell type found in the immediate proximity of tumors. Furthermore, these cells do not undergo senescence, allowing longer experiments to be conducted as compared to other commonly used cell types [27]. Toxicity assessment was based on previously established methods [7, 28]. Briefly, RPE-1 cells were plated in 96-well plates at a density of 100 cells/mm^2^ and allowed to adhere for 24 h at 37 °C, 5 % CO_2_. Cells were incubated with media containing the PEGylated nanoparticles at concentrations ranging from 5–100 μg/mL. These concentrations represent the maximum anticipated amount of nanoparticle exposure to normal cells based on tumor accumulation due to systemic and local injections, as previously reported [28]. Nanoparticles were incubated with cells for 24 h at 37 °C, 5 % CO_2_. Cell toxicity was analyzed using both the Celltiter-glo® cell viability assay (Promega) and the lactase dehydrogenase (LDH) cytotoxicity assay (Piercenet). Celltiter-glo quantifies the concentration of adenosine triphosphate (ATP) and thus number of metabolically active cells post nanoparticle exposure; the LDH assay measures the amount of lactate dehydrogenase released by damaged cells into the media. Experimental controls were defined as plain cells in media and pure media with and without nanoparticles. Toxicity assessment and calculations were conducted according to the manufacturers’ protocols. Statistical analysis was performed using JMP analysis of variance (ANOVA) to compare results between groups. Tukey’s post hoc test was used in conjunction with ANOVA for further analysis. Differences were recorded to be statistically significant at *p* < 0.05. All errors are given as standard deviations.

## Results and Discussion

### Structure, Formation, and Elemental Composition of Icosahedra GGS NP

**Fig. 1:**
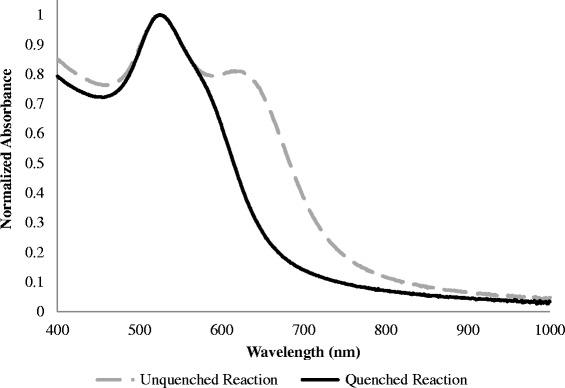
Microwave-assisted nanoparticle synthesis. The quenched reaction resulted in the formation of only gold nuclei (520-nm peak) indicative of small gold colloid. The unquenched reaction produced an additional peak (620 nm) originally thought to be associated with the formation of gold-coated gold sulfide nanoparticles

**Fig. 2:**
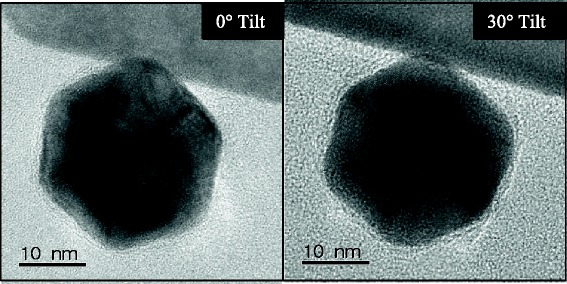
TEM of icosahedral nanoparticles with 30° tomography tilt angle. With the stage tilt, different facets become visible, in addition to the change in crystal orientation, confirming the 3D structure of the icosahedral nanoparticles. *Scale bars* represent 10 nm

Original models based upon the Mie theory have suggested that the NIR SPR associated with GGS NP are due to a hexagonal core-shell structure [10]. More recent reports have hypothesized that the NIR peak should actually be attributed to gold nanoparticle aggregates, thus suggesting that the GGS NP are, therefore, not comprised of a core-shell configuration [12, 13]. To further elucidate the formation of GGS NP, we first used conventional formulations with microwave irradiation, halting the reaction (via quenching) immediately after nucleation was initiated. Microwave irradiation has been used extensively by many researchers to accelerate nucleation [29]. The quenched reaction formed a monodisperse population of small gold colloid, as evidenced by the single absorption peak at 520 nm (Fig. 1). By comparison, when the same microwave-assisted reaction was allowed to proceed for 1 h at room temperature, the same peak at 520 nm remains and a second, red-shifted (~620 nm) peak becomes evident. Previously, it was stated that this second peak was due to the formation of the gold-coated gold sulfide nanoparticles [10]. However, high-resolution TEM (HR-TEM) images of the resulting particles show homogeneous icosahedral nanostructures with defined facets (Fig. 2). This conclusion is further confirmed by the tilting of the TEM stage; as the angle changes, different facets become focused and defined, thus confirming a 3D icosahedra structure with no evidence of a core-shell configuration [30]. Furthermore, the second absorption peak at 620 nm is not consistent with reported spectra for gold-coated gold-sulfide nanocolloids, found to be near 790 nm [4]. These results collectively suggest that no gold sulfide nanoparticles are being produced at this stage but rather the icosahedral species of the conventional one-pot method.

**Fig. 3:**
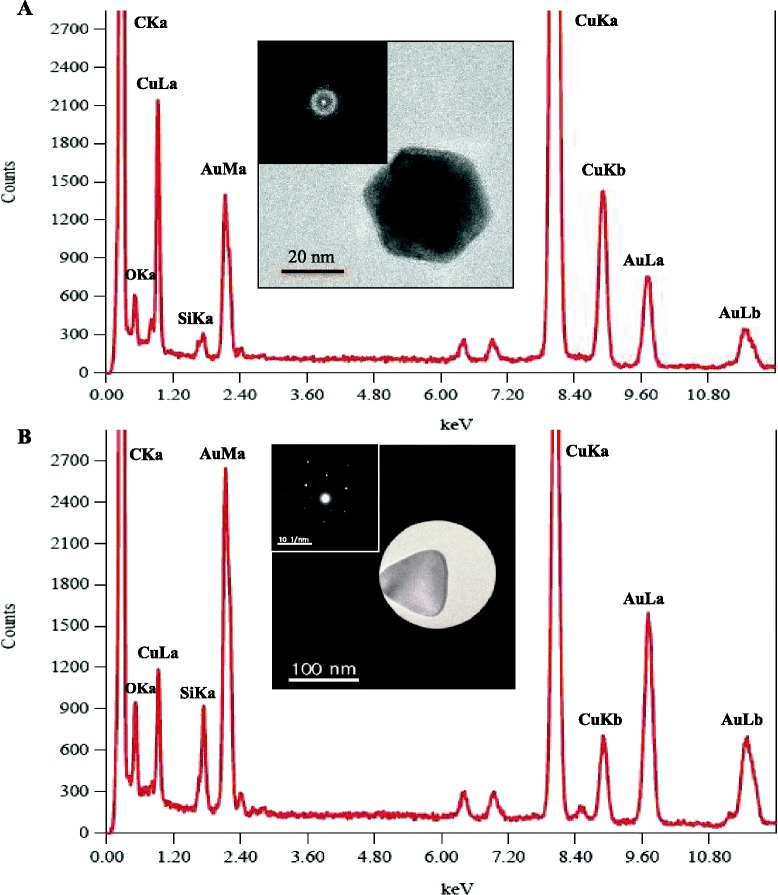
EDS spectra of a single icosahedral particle with corresponding FFT insert (**a**) and a single nanoprism with corresponding SAED insert (**b**). EDS displays evidence of a predominately gold sample indicated by the absence of a sulfur peak (2.307 keV). The FFT shows a spacing of 2.35 Å, consistent with [111] FCC gold. The SAED shows spacing of 1.424 and 2.5 Å, corresponding with crystal lattice spacing and forbidden reflections of gold

We next used standard GGS NP fabrication procedures and determined the elemental composition of both icosahedra particles and nanoplates using EDS, FFT, and SAED analysis (*n* = 4 particles per analysis). These two particle types were analyzed since the standard GGS NP fabrication procedure results mostly in both particle populations. Based on this analysis, the nanoplates and icosahedra particles produced identical spectra consistent with face center cubic (FCC) gold (Fig. 3). The largest peaks are carbon (C) and copper (Cu), components of the TEM grid, and iron (Fe) and cobalt (Co), which are constituents of the TEM grid holder. Outside of the extraneous peaks, the peaks of interest are all derivatives of gold with no evidence of sulfur, which has a characteristic EDS peak at 2.307 keV. If sulfur was present in the sample, it is present at less than one part per billion, providing no significant contribution to the particle structure nor role in its bulk material properties. SAED and FFT analyses showed the predominant lattice spacing correspond to FCC [111] and [100] Au, with some forbidden reflections that can be indexed as ½[422] FCC Au [31]. Forbidden reflections are common in highly faceted gold nanoparticles with an artifact that results in a lattice spacing of 2.5 Å. These characterization results indicate that both nanoparticle populations (icosahedra and nanoplates) are predominately gold with negligible amounts of sulfur. These results support previous claims that sodium thiosulfate acts as both a reducing agent and a shape-directing agent, facilitating growth of nanoparticles predominately into their preferred highly faceted multiple-twinned structure with mainly [111] and [100] facets [32]. Formation of nanoplates is most likely due to insufficient amounts of sodium thiosulfate as well as nucleation and growth occurring simultaneously.

### Seed-Mediated GGS NP Synthesis

**Fig. 4:**
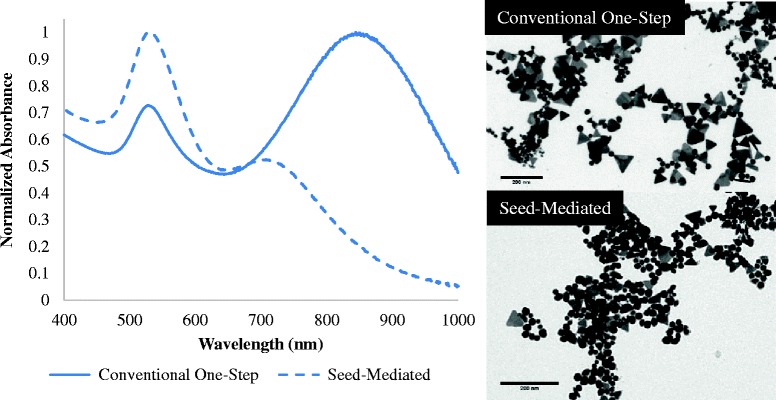
Seed-mediated growth of gold-gold sulfide nanoparticles. Seed-mediated growth of particles resulted in suppression and blue-shifting of the NIR peak. Corresponding TEM image shows predominantly icosahedral particles with reduced nanoplate formation and size (<50 nm) as compared with conventional one-step synthesis. *Scale bars* represent 200 nm

To improve the monodispersity of GGS NP, we created GGS NP based on seed-mediated growth, as this method allows for greater synthesis control. Seed-mediated growth is often used to increase control over the reaction conditions due to the separation of nucleation and growth steps [33]. Small gold seeds (5 nm) were successfully synthesized as previously reported [24]. The optimal seed-mediated condition involved incubation of sulfide with 5-nm gold colloid for 24 h and then adding additional gold salt. Colloid that was not aged resulted in globular, ill-defined particles, indicating the importance of sulfide in determining the overall shape of the particles (data not shown). The seed-mediated method resulted in a NIR peak located at ~717 nm (Fig. 4), which was much lower and blue-shifted compared to the conventional synthesis which results in a NIR peak ~800 nm. Corresponding TEM images show that the distribution of the nanoplates resulting from seed-mediated synthesis was significantly reduced (by ~50 %) compared to the conventional one-step synthesis method. As shown in Fig. 4, the seed-mediated sample consisted predominately of icosahedral nanoparticles, which made up approximately 82 % of the entire sample. While nanoplates were still formed using this seed-mediated method, these particles were smaller (~50 nm) and fewer in number than that produced by the conventional method. This provided the first indication that the icosahedral nanoparticles were not the species contributing to the NIR peak, which is seen with the conventional one-step synthesis method. While the seed-mediated method resulted in a minor NIR peak (~717 nm), TEM results suggest that the 717-nm peak may be due to the inclusion of smaller and less concentrated nanoplates. In a conventional reaction, the NIR peak is a result of the aggregation between icosahedral and nanoplate particles, producing one combined peak near 800 nm [11, 12]. These results indicate that the corresponding proportion of icosahedral to nanoplate phenotypes is responsible for the blue- or red-shifting of the NIR peak.

### Elucidation of Temperature Effects on NIR-Contributing GGS NP Species

**Fig. 5:**
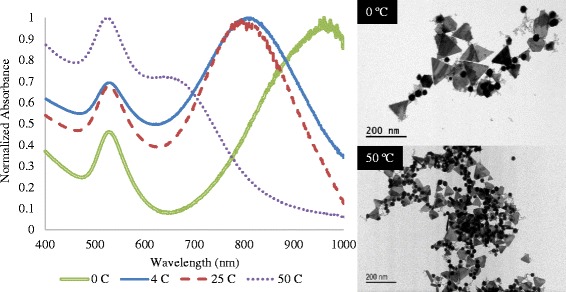
Effect of temperature on conventional one-step gold-gold sulfide synthesis. Room temperature reaction provides characteristic NIR peak (800 nm) and minimal gold colloid peak (520 nm) with the production of polydisperse nanoparticles. At lower temperatures (0 °C), the presence of larger gold nanoplates results in red-shifting and broadening of the NIR peak. At higher temperatures (50 °C), the dramatic increase in icosahedral nanoparticles results in diminished NIR peak. *Scale bars* represent 200 nm

We further examined the effects of temperature alterations to control conventional reaction kinetics to confirm the NIR-contributing species. The effect of varying fabrication temperature (from 0 to 50 °C) on the SPR is shown in Fig. 5. Traditional fabrication of GGS NP occurs at room temperature (25 °C). This synthesis condition results in an optimal SPR peak for photothermal therapy (~800 nm) yet comprises significant shape variations, consistent with previously published results [5]. At a higher fabrication temperature of 50 °C, the traditional NIR peak is replaced by a reduced peak at ~650 nm with a dominant 520-nm peak, suggesting that higher temperatures yield less of the NIR-contributing species. At high temperatures, the gold precursor experiences an increased nucleation rate, increasing the reaction kinetics and reduction rate [34–36]. Thus, the formed particles are likely to be smaller and icosahedral in shape, due to a reduced growth rate as a result of the increased reduction [34]. Conversely, for considerably lower temperatures (0 °C), the NIR peak red shifts and broadens beyond the 900-nm range, with the absorbance peak at 520 nm decreasing in intensity. This data is in agreement with results presented by James et al. [22] which showed that wavelength increased (red-shifted) with decreasing temperatures to tune the peak absorbance of gold nanoplates beyond 900 nm. The larger particles (gold nanoplates) may be formed as a result of the decreased rate of reduction at these lower temperatures. TEM images reveal the predominance and increased size of nanoplates synthesized at 0 °C compared to a dramatic increase in icosahedral nanoparticle formation at 50 °C. While nanoplates are still formed at 50 °C, the predominance of icosahedral nanoparticles likely results in the suppression of the characteristic gold nanoplate NIR plasmon resonance observed at room temperature. This study suggests that the presence of the nanoplates contributes substantially to the NIR SPR wavelength and intensity using the conventional one-step GGS NP synthesis.

### Size-Selective Separation of Polydisperse Samples

**Fig. 6:**
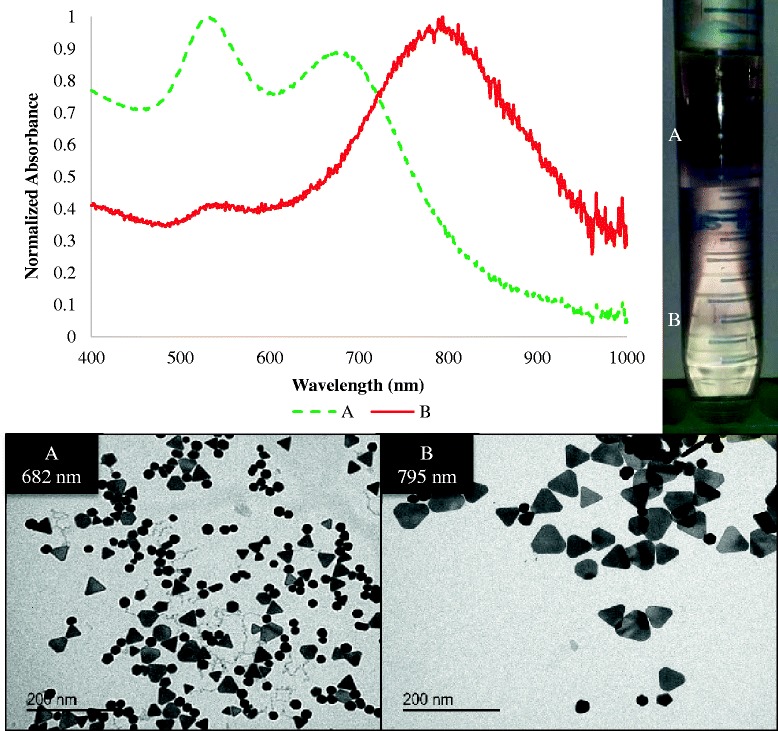
Glycerol separation of nanoparticles. *Fraction A* consists of predominately icosahedral nanoparticles with few nanoplates. This fraction has two absorption peaks, the maximum at 520 nm and a smaller peak at 682 nm. *Fraction B* is composed predominately of nanoplates with a red-shifted SPR peak at 795 nm. It is noted that nanoplates found in *A* are much smaller than those found in *B. Scale bars* represent 200 nm

To support our hypothesis that the nanoplates are the only particles contributing to the NIR peak, we used microfiltration to isolate different nanoparticle populations. Previous studies have traditionally purified NIR-gold nanoparticle mixtures through multiple centrifugation steps and more recently utilized regenerated cellulose membranes (RCM) in a novel synthesis process named “DiaSynth” [22, 37]. In these studies, we wanted to create a homogenous nanoparticle suspension by using a rapid filtration method following a conventional one-pot synthesis, for the confirmation of the NIR-contributing species. To isolate the different nanoparticle subtypes, we employed both glycerol density separation and sequential micron pore filtration. Glycerol density separation has been widely published in the literature and has been used for many different nanoparticle applications, as it allows the separation of bare particles [25]. However, residual glycerol must be removed after separation, requiring multiple wash cycles. For the GGS NP, the optimal conditions for particle separation in glycerol were 40 min at 3200 *g* and 40 °C using a 30–90 % concentration gradient. This resulted in clean separation bands with minimal streaking, as shown in Fig. 6. Samples were carefully handled in order to remove the desired nanoparticle subsets and avoid the blending of bands. This process was difficult to reproduce, as any disruption in sample preparation would inappropriately mix the bands. In this regard, extraction of the bands was prone to manual error, increasing the likelihood of cross-over between separated samples. Density filtration demonstrated that the largest particles were predominately nanoplates, whose corresponding spectra revealed a NIR peak near 795 nm. Equally, small density bands consisted primarily of icosahedra-shaped nanoparticles, which were previously suggested as the NIR-contributing species, whose plasmon peak was approximately 680 nm. While nanoplates were present in fraction A, as previously suggested, the predominance of the icosahedral nanoparticles likely results in a relative reduced plasmon peak.

**Fig. 7:**
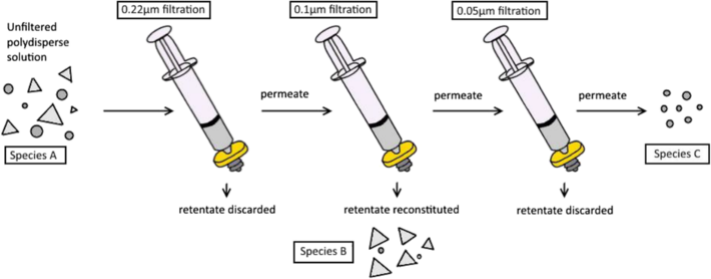
Microfiltration process of polydisperse samples. Samples of interest, *species B* and *C* were collected via sequential filtration steps. *Species B* includes all particles retained by the 0.1-μm filter. *Species C* contains particles which passed through the 0.05-μm filter

**Fig. 8:**
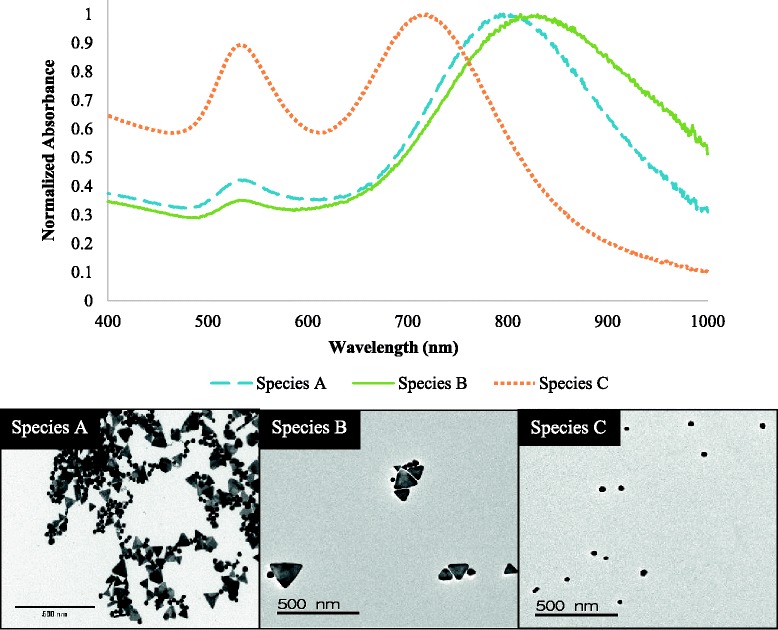
Selective size filtration of polydisperse nanoparticles. Sequential filtering results in blue-shifting of the NIR peak and removal of the larger nanoplates. *Scale bars* shown represent 500 nm

In addition to glycerol density separation, we performed sequential micron pore filtration. This technique was used to filter both bare and PEGylated particles, both of which passed efficiently through the filters (Fig. 7). To recover the retained nanoparticles, the filters were sonicated in a water bath to release captured particles. This simple technique resulted in the quickest, most efficient separation of nanoparticles compared to other methods. The two filtered samples of interest were those recovered from the 0.1-μm filter and those that passed through the 0.05-μm filter, species B and C, respectively. These two filtered species were compared to the polydisperse sample produced via the conventional one-pot synthesis, species A. As shown in Fig. 8, species C has a SPR peak at 705 nm resembling that of a primarily icosahedral particle sample theorized from Fig. 1. Species B is red-shifted with a NIR SPR peak at 834 nm. TEM images analyzed through ImageJ demonstrated that species C, the most blue-shifted fraction, consisted predominately of icosahedral particles (~77 %) while species B, the most red-shifted fraction consisted largely of nanoplates (~57 %). These results, detailed in Table 1, support the hypothesis that nanoplates are the predominant species contributing to the NIR peak. This is supported by Millstone et al. who have identified nanoprisms as typically having SPRs throughout the visible and NIR regions by means of controlling prism thickness, edge length, and vertex sharpness [2]. While we primarily investigate the influence of nanoparticle composition on the resulting SPR, Millstone et al. draw attention to the blue- or red-shifting of nanoplate SPRs as a result of adjusting structural variables (edge length, thickness, and degree of truncation) [2]. The size separation of the polydisperse nanoparticle samples and examination of corresponding UV–vis spectra in tandem with TEM images confirms the contribution of the nanoplates to the NIR SPR, not the icosahedra nanoparticles.

**Table 1:** Nanoparticle size, NIR peak, and nanoplate composition

Sample	Diameter size (nm)	NIR peak λ (nm)	Nanoplate composition (%)
Species A	10–300	800	26
Species B	100–220	834	57
Species C	<50	705	23

### GGS NP Toxicity Studies

Nanoparticle size effects are well known to greatly affect cellular toxicity as well as corresponding biodistribution profiles [38]. Studies examining the effect of gold nanoparticle size on biodistribution have shown that small particles (10 nm) predominately distribute and localize in the blood, liver, kidney, heart, and brain, whereas larger particles (50–250 nm) were only detected in the blood, liver, and spleen [39]. These size effects are confounded by additional shape variations, which have different charges, defects, and surface areas compared to uniform samples, resulting in unpredictable behaviors in vivo [40]. Bismuth selenide (Bi_2_Se_3_) nanoplates, 60 nm in diameter, were previously shown to accumulate in the liver, spleen, and kidney [41]. Previous studies have also demonstrated the preferential uptake in macrophages of spherical particles over those with high aspect ratios [42].

**Fig. 9:**
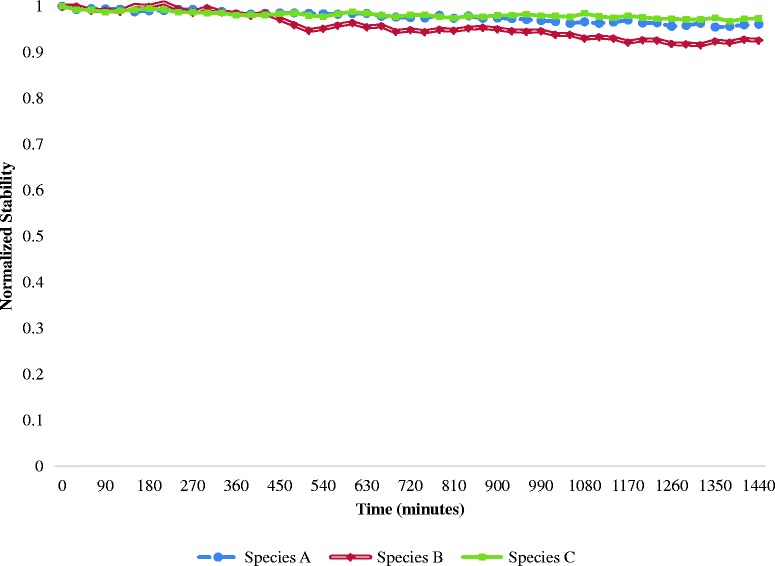
Stability of the three filtered species of nanoparticles in DMEM-F12 media. *Species C* was found to be the most stable and *species B* the least stable. This effect can be attributed to the settling of the larger particles due to gravity

**Fig. 10:**
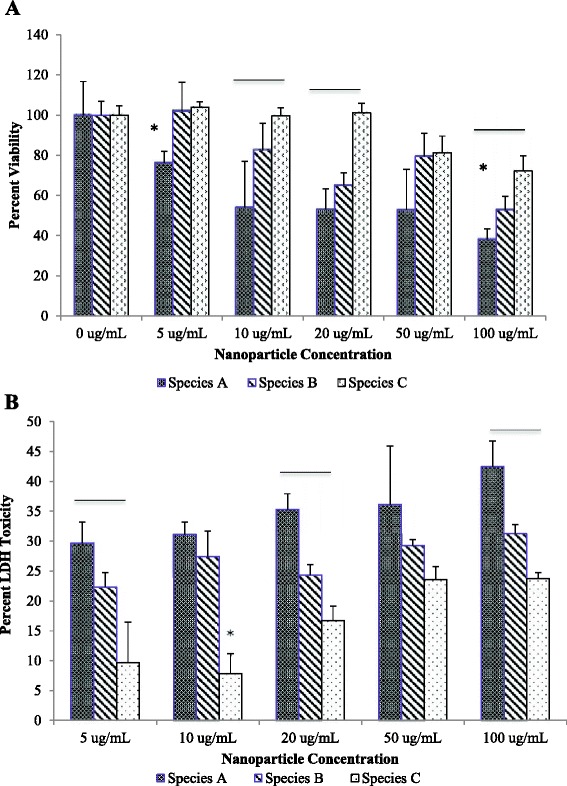
Cytotoxicity of nanoparticles to RPE-1 cells: Celltiter-glo (**a**) and LDH assay (**b**). The non-filtered, polydisperse particles displayed the most cytotoxicity while the more homogeneous and smaller particles were shown to be the least toxic. *Line* above samples indicates that all three values are statistically significant (*p* < 0.05). *Asterisk* denotes one sample which is statistically different from the other two (*p* < 0.05). *Error bars* represent standard deviation

To determine the impact of sample purification on cytotoxicity, the aforementioned three different nanoparticle populations (species A, B, and C) were evaluated. Samples were prepared at comparable concentrations ranging from 5 to 100 μg/mL. Prior to conducting experiments, particle stability in culture media was analyzed. Toxicity effects can be skewed by the effect of nanoparticles settling in the media over time. While all samples were stable for 24 h, the nanoplate fraction (species B) showed the least stability, as evidenced by the greater temporal decrease in the maximum peak absorbance (Fig. 9). This effect is expected due to the larger size, and increased surface area, of the nanoplates, resulting in increased settling and possible protein adsorption. However, this effect only resulted in 6 % loss in stability. Using both cell viability and LDH assays, our analyses showed that species A resulted in the greatest cytotoxicity, whereas species C resulted in the least toxicity (Fig. 10). While previous studies have stated that smaller particles are more toxic than larger nanoparticles of the same shape due to enhanced surface area, shape effects tend to dominate [38, 34]. Specifically, nanoplates are reportedly more toxic than spherical particles due to their propensity for surface defects [40]. However, our results suggest that polydispersity (with the most variation in both size and shape) may result in the greatest cytotoxicity. While we demonstrate that variations in both shape and size impact toxicity using simplified 2D assays, these effects are also likely to be exacerbated when moving into 3D assays and animal models. Sample polydispersity not only affects cellular uptake and toxicity but likely provides undesirable variations in biodistribution in vivo [42]. These findings further dictate the need to produce well characterized and monodisperse samples prior to advancing to clinical nanomedicine trials.

## Conclusions

Conventional one-step synthesis of NIR GGS NP was originally proposed as having an icosahedral, sulfide core-gold shell nanostructure, yet fabrication results in a polydisperse solution of nanoplates, icosahedral nanoparticles, and irregularly shaped asymmetric nanoparticles. Subsequent size-selective separation and compositional analysis reveal that the elemental composition of these particles is FCC gold and that nanoplates are the predominant NIR-contributing species, not the icosahedral nanoparticles as originally hypothesized. Here, we optimized two filtration methods, both of which facilitated nanoparticle separation, with microfiltration providing the most reproducible and rapid fractionation. Importantly, the original polydisperse samples produced by the conventional one-step reduction of chloroauric acid by sodium thiosulfate resulted in enhanced cellular toxicity in vitro compared to more purified samples. While further work is needed to elucidate the toxic properties of nanomaterials universally, this study highlights the need to fully characterize and optimize nanomaterials before translating into more complex and biologically relevant animal and pre-clinical models. This upstream analysis will expedite the development of highly promising translational nanomaterials for in vivo applications and ultimate FDA approval.

## Abbreviations

NIR: near-infrared
TEM: transmission electron microscopy
EDS: energy dispersive x-ray spectroscopy
FCC: face center cubic
SPR: surface plasmon resonance
GGS NP: gold-gold sulfide nanoparticle
FDA: Food and Drug Administration
OD: optical density
SAED: selected area electron diffraction
FFT: fast Fourier transform
RPE-1: hTERT-RPE-1
LDH: lactose dehydrogenase
ATP: adenosine triphosphate
ANOVA: analysis of variance
